# Variation of PPARG Expression in Chemotherapy-Sensitive Patients of Hypopharyngeal Squamous Cell Carcinoma

**DOI:** 10.1155/2021/5525091

**Published:** 2021-05-17

**Authors:** Meng Lian, Yong Tao, Jiaming Chen, Xixi Shen, Lizhen Hou, Shaolong Cao, Jugao Fang

**Affiliations:** ^1^Department of Otorhinolaryngology Head and Neck Surgery, Beijing Tongren Hospital, Capital Medical University, Beijing 100730, China; ^2^Department of Pharmacy, Liaocheng Third People's Hospital, Liaocheng, Shandong 252000, China; ^3^Department of Bioinformatics and Computational Biology, The University of Texas MD Anderson Cancer Center, Houston, TX 77030, USA

## Abstract

Our previous study showed that the upregulation of peroxisome proliferator-activated receptor gamma (PPARG) could promote chemosensitivity of hypopharyngeal squamous cell carcinoma (HSCC) in chemotherapeutic treatments. Here, we acquired two more independent expression data of PPARG to validate the expression levels of PPARG in chemotherapy-sensitive patients (CSP) and its individualized variations compared to chemotherapy-non-sensitive patients (CNSP). Our results showed that overall PPARG expression was mildly downregulated (log fold change = −0.55; *p* value = 0.42; overexpression in three CSPs and reduced expression in four CSPs), which was not consistent with previous results (log fold change = 0.50; *p* = 0.22; overexpression in nine CSPs and reduced expression in three CSPs). Both studies indicated that PPARG expression variation was significantly associated with the Tumor-Node-Metastasis (TNM) stage (*p* = 7.45*e* − 7 and 6.50*e* − 4, for the first and second studies, respectively), which was used as one of the predictors of chemosensitivity. The new dataset analysis revealed 51 genes with significant gene expression changes in CSPs (LFC > 1 or <-1; *p* value < 0.01), and two of them (TMEM45A and RBP1) demonstrated strong coexpression with PPARG (Pearson correlation coefficient > 0.6 or <-0.6). There were 21 significant genes in the data from the first study, with no significant association with PPARG and no overlap with the 51 genes revealed in this study. Our results support the connection between PPARG and chemosensitivity in HSCC tumor cells. However, significant PPARG variation exists in CSPs, which may be influenced by multiple factors, including the TNM stage.

## 1. Introduction

Hypopharyngeal squamous cell carcinoma (HSCC) accounts for about 5% of head and neck tumors and is one of the top human malignancies in Europe and the United States [1]. Each year, HSCC causes about 10 cases per million people in the world, with more than 160,000 new cases and 83,000 deaths [2, 3]. Due to the poor survival rate and the devastating impacts on swallowing and speech, the administration of HSCC remains one of the most challenging topics [4]. Patients with HSCC are usually treated with chemoradiotherapy to preserve the organ and its function [5]. PPARG (peroxisome proliferator-activated receptor gamma) is a protein-coding gene, which has been suggested to improve chemosensitivity in human carcinomas, including HSCC [6–9].

Our previous study showed that elevated PPARG expression could drive multiple molecules to increase the chemosensitivity of multiple squamous carcinoma cells [9]. For example, the activation of PPARG was shown to increase the expression of BMP6, BMP7, and NME1 [10, 11], which was positively related to the chemosensitivity of multiple squamous carcinoma c5ells [12–14]. Moreover, PPARG has been suggested to depress the expression of multiple chemosensitivity inhibitors, such as TERT, CFTR, and EGR1 [9], which form another type of pathway for the chemosensitivity promotion role of PPARG [15–17].

Our previous study also showed that PPARG could demonstrate increased expression levels in HSCC chemotherapy-sensitive patients (CSP) compared to chemotherapy-non-sensitive patients (CNSP) [9], supporting the role of PPARG in chemosensitivity promotion. However, a significant variance was observed among the individuals within the CSP group, resulting in a mild overall expression change. In this study, we explored the expression changes of PPARG in the CSP group by acquiring further expression data and tested the potential influence of multiple clinical parameters. Our results confirmed the association between PPARG and chemosensitivity in HSCC patients as well as its strong expression variance among individual HSCC subjects, which suggested that PPARG may be among multiple factors that influence the chemotherapy sensitivity of HSCC patients.

## 2. Materials and Methods

### 2.1. Patient Recruitment and Specimen Selection

In our previous study, microarray expression data of 21 HSCC patients were acquired, including 12 CSPs and 9 CNSPs [9]. These patients were undergoing induction chemotherapy for primary HSCC. We submitted our data to Gene Expression Omnibus (GEO; https://www.ncbi.nlm.nih.gov/geo/) with GEO ID GSE85608. Following the same data acquisition workflow, we acquired the expression data of another 11 HSCC patients, which is also available on GEO (GEO ID GSE85607). We provided the clinical features of these HSCC patients in Tables 1 and 2, respectively. For more details of the two datasets, please refer to https://www.ncbi.nlm.nih.gov/geo/query/acc.cgi?acc=GSE85608 and https://www.ncbi.nlm.nih.gov/geo/query/acc.cgi?acc=GSE85607, respectively.

### 2.2. PPARG Expression and Potential Influential Factors

For the two datasets, we renalyzed the expression levels of PPARG at probe ILMN_1800225 (probe sequence: CCTGAGCCACTGCCAACATTTCCCTTCTTCCAGTTGCACTATTCTGAGGG), focusing on its variation and potential influential factors. We first compared the expression levels in the CSP group and CNSP group in terms of log fold change (LFC) using one-way ANOVA. Then, we employed a multiple linear regression (MLR) model to study the potential connection between PPARG levels and multiple clinical parameters, including age, TNM stage, histologic differentiation (HD) degree, and chemotherapy response (CR). For the nonnumeric variables, the original string value was changed to a numeric value by indexing different values. Beta values, 95% confidence intervals of beta values, and parameter significance in terms of *p* values were reported. All the analyses were performed using MATLAB (version R2017a).

### 2.3. Coexpression Analysis

To explore the coinfluential genes that play roles in the CSPs of HSCC disease, we first identified the genes that demonstrated a significant change in the CSP group compared with the CNSP group (LFC > 1 or <-1 and *p* < 0.01) using one-way ANOVA (function “anaova1” in the statistics toolbox of MATLAB). Then, we calculated the pairwise linear correlation between PPARG expression and that of these significant genes (function “corr” in the statistics toolbox of MATLAB). The RHO (Pearson's correlation coefficient) value was used to evaluate the strength of the coexpression: (1) strong correlation: abs(RHO) ∈ [0.6, 1]; (2) medium correlation: abs(RHO) ∈ [0.4, 0.6]; (3) weak correlation: abs(RHO) ∈ [0.2, 0.4]; and (4) no correlation: abs(RHO) < 0.2. Here, abs(RHO) refers to the absolute value of RHO. All analyses have been conducted using MATLAB (version R2020a).

### 2.4. Pathway Analysis

To explore the functionality of the significant genes in the CSP group that also presented coexpression with PPARG (abs(RHO) ≥ 0.2), we conducted Fisher's exact test-based pathway enrichment analysis (PEA) (https://david.ncifcrf.gov/content.jsp?file=functional_annotation.html#fisher) against Gene Ontology (GO) [18]. In addition, a literature-based network analysis was conducted using Pathway Studio (http://www.pathwaystudio.com) to uncover potential cofunctional genes of PPARG. For the detailed instructions regarding network analysis, please refer to the supporting materials at https://supportcontent.elsevier.com/Support%20Hub/Pathway%20Studio/Network%20Builder%20basic%20_Interactive%20NB%20v114.pdf.

## 3. Results

### 3.1. PPARG Expression in the CSP Group

**Figure 1:**
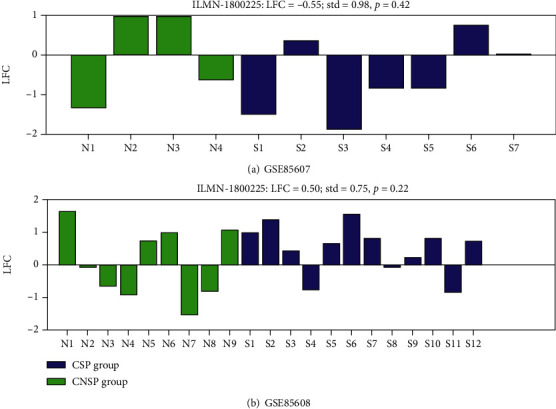
PPARG expression in terms of log fold change (LFC) in chemotherapy-sensitive patients (CSP) among all HSCC patients: (a) PPARG expression of HSCC patients in the dataset GSE85607; (b) PPARG expression of HSCC patients in the dataset GSE85608.

For the two datasets analyzed, we presented the expression of PPARG for all HSCC patients in Figure 1, including both CSPs and CNSPs. In dataset GSE85608, PPARG presented an overall increased expression in the CSP group compared to the CNSP group (LFC = 0.50; *p* = 0.22; see Figure 1(b)). However, in dataset GSE85607, PPARG presented an overall reduced expression (LFC = −0.55; *p* = 0.42), with more patients presented decreased expression than overexpression (four vs. three; see Figure 1(a)). Moreover, there were also significant variances among the CNSP group (green bars in Figures 1(a) and 1(b)). These results suggested that there were influential factors causing the variation of PPARG expression among HSCC patients, which is worthy of further study.

### 3.2. TNM Stage and PPARG

**Figure 2:**
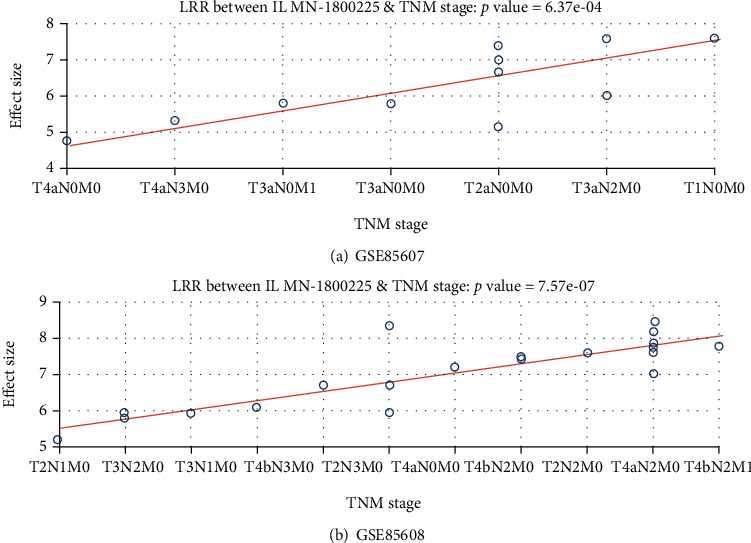
Association between PPARG expression and TNM stage in HSCC patients: (a) association plot by using data of HSCC patients in the dataset GSE85607; (b) association plot by using data of HSCC patients in the dataset GSE85608. The expression levels were log2-transferred.

MLR results by using data from both GSE85607 and GSE85608 showed that the expression levels of PPARG were significantly associated with the TNM stage (*p* value = 6.37*e* − 4 and 7.57*e* − 7 for GSE85607 and GSE85608, respectively), as shown in Figure 2. However, due to the limited sample size, TNM stages were not well matched among samples within the two datasets. More data with a larger sample size is needed to better understand the linkage between TNM stage and PPARG expression levels.

To note, the *p* values for the beta factor of chemotherapy response (CR) did not reach the significance level (*p* value = 0.16 and 0.26 for GSE85607 and GSE85608, respectively). This was consistent with the mild overall expression changes of PPARG in the CSP group compared with the CNSP group.

Moreover, the other two parameters, namely, age and histologic differentiation (HD) degree, were not significant factors influencing PPARG expression levels (*p* value > 0.42). We presented the detailed results in Supplementary Material PPARG_HSCC_CR➔MLR_GSE85607 and MLR_GSE85608. The Supplementary Material PPARG_HSCC_CR is a multiworksheet Excel file that contains additional results of this study, including the MLR analysis results, ANOVA and correlation analysis results, gene set enrichment analysis results, and references for the network analysis.

### 3.3. Significant Genes and Coexpression Analysis

For dataset GSE85607, 51 significant genes (LFC > 1 or <-1; *p* < 0.01) were identified in the comparison between CSP and CNSP groups. The number of significant genes for dataset GSE85608 was 21. To note, there was no overlap between the two groups of significant genes identified, indicating the different overall genomic variances among the HSCC patients recruited in the two studies. We provided the analysis statistics in Supplementary Material PPARG_HSCC_CR➔Corr_GSE85607 and Corr_GSE85608.

Coexpression analysis showed that, in dataset GSE85607, PPARG was strongly correlated with two genes (RBP1 and TMEM45A) and also presented a weak to moderate correlation with other 19 other genes. Interestingly, PPARG was negatively correlated with the genes that demonstrated overexpression in the CSP group and a positive correlation with downregulated genes. Please see PPARG_HSCC_CR➔Corr_GSE85607 for details. This partially explains the overall downregulation in dataset GSE85607.

In contrast, PPARG was only moderately correlated with one gene (MYOM3; RHO = −0.46) that presented downregulation in the CSP group and weak correlation with seven other genes (see PPARG_HSCC_CR➔Corr_GSE85608). RHO here refers to Pearson's correlation coefficients. Moreover, the genes that showed overexpression were mostly positively correlated with PPARG, while those with reduced expression were all negatively correlated with PPARG. These results partially explain the overall increased PPARG expression in the CSP group of GSE85608.

### 3.4. Pathway Enrichment Analysis and Network Analysis

For the 29 CSP-significant PPARG coexpressed genes from both datasets, we conducted a PEA against the Gene Ontology (GO). However, none of these pathways passed the false discovery rate (FDR) with *q* value = 0.05. We presented the details in PPARG_HSCC_CR➔PEA. Our results suggested that these genes may not be closely linked to each other in terms of biological functionality.

**Figure 3:**
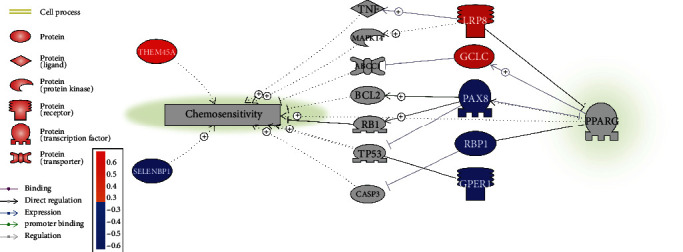
Cofunctional genes of PPARG to play roles in the chemosensitivity in HSCC patients. Nodes in red were positively correlated with PPARG in terms of expression; nodes in blue were negatively correlated with PPARG.

Literature-based data mining showed that seven out of the 29 CSP-significant genes were linked to PPARG and chemosensitivity, as shown in Figure 3. The network was built based on a total of 334 references, which were provided in PPARG_HSCC_CR➔Ref4Network, including titles and sentences where a relationship has been identified.

It was worthy of mentioning that PPARG presented a positive correlation with two out of three chemosensitivity promoters (LRP8 and GCLC) and a negative correlation with the three chemosensitivity inhibitors (PAX8, GPER, and RBP1), which supports the chemosensitivity promotion role of PPARG in HSCC patients that was proposed in our previous studies.

## 4. Discussion

Our previous study suggested the chemosensitivity promotion role of PPARG in HSCC patients and also indicated the variation of PPARG expression levels among individual HSCC subjects. In this study, we confirmed our previous findings by using two independent expression data of HSCC chemotherapy-sensitive and nonsensitive patients and tested multiple potential influential factors for PPARG expression. Our results suggested that the expression of PPARG was strongly influenced by TNM stage and was correlated with multiple genes that show significant differential expression in CSP/CNSP comparison.

The expression of PPARG was not consistent in the two datasets. Specifically, most CSPs (9 out of 12) in GSE85608 showed overexpression, resulting in overall increased PPARG expression levels in the CSP group. However, in GSE85607, more CSPs (4 out of 7) presented decreased expression, leading to reduced expression levels of PPARG, as shown in Figure 1. These results suggested the variation of PPARG expression in HSCC patients that is worthy of further study.

MLR results showed that PPARG expression in HSCC patients was significantly linked to the TNM stage (Figure 2), which has been implicated as one of the clinical features to predict chemosensitivity [19]. However, due to the limited sample size, the TNM stage was not well matched within the two datasets, which made it difficult to explain the influence of different TNM stages on PPARG expression. Specifically, the highest PPARG expression level was identified in an HSCC patient in the stage of T1N0M0 (Figure 2(a)), which represented a stage that the tumor development was at its earliest stage, with no significant influence on the regional lymph nodes and no metastasis. The lowest PPARG expression was observed from an HSCC patient in a stage of T4aN0M0, which means that the tumor size and extension of the primary tumor were at the late stage, but with no influence on regional lymph nodes and no metastasis. In contrast, for dataset GSE85608, the highest PPARG expression was observed in an HSCC patient at the stage of T4aN2M1, which means that the development of the tumor in this patient was at its late stage with moderate influence on the regional lymph nodes and early appearance of metastasis. Studies with a larger sample size covering all different TNM stages should be conducted to fully understand the correlation between PPARG expression in HSCC patients and their TNM stages.

Consistent with the mild expression changes of PPARG in the CSP group compared with the CNSP group, chemotherapy response (CR) was moderately correlated with the expression of PPARG (*p* value = 0.16 and 0.26 for GSE85607 and GSE85608, respectively). Moreover, age and histologic differentiation (HD) degree were shown to be nonsignificant factors for PPARG expression levels (*p* value > 0.42). Please refer to PPARG_HSCC_CR➔MLR_GSE85607 and MLR_GSE85608 for more details of the MLR results.

The significant variation of PPARG in HSCC CSPs suggested that there were other factors cofunctioning with PPARG to influence the chemosensitivity of HSCC patients. However, among the genes that showed significant expression variance in CSP/SNSP comparison, only a small portion (9 out of 72 genes) showed a moderate to strong correlation with that of PPARG (absolute value of RHO > 0.4). Please refer to PPARG_HSCC_CR➔Corr_GSE85607 and Corr_GSE85608 for the details of coexpression analysis. Among these genes, 7 genes were implicated to have a relation with PPARG and chemosensitivity, as shown in Figure 3. These genes could be the cofunctional factors that work with PPARG to influence the chemosensitivity of HSCC patients. For instance, LRP8 was shown to activate TNF and MARK14 [20, 21], which are promoters of chemosensitivity [22, 23]. In addition, overexpression of GCLC mRNA suppresses the expression of MRP1 [24], which in turn could improve the chemosensitivity in lung cancer patients [25]. The positive correlation between PPARG and LRP8 and GCLC indicated the cofunctionality of these genes and PPARG in the chemosensitivity promotion of HSCC patients. On the other hand, PPARG was negatively correlated with multiple inhibitors of chemosensitivity, including PAX8, GPER1, and RBP1. Yu et al. showed that the blockage of GPER/ABCG2 signaling could be a potential target for enhancing the chemosensitivity of breast cancer patients [26]. Chen et al. showed that RBP1 gene transfection could significantly reverse L5-induced increases in CASP3 [27], while overexpression of CASP3 has been shown to enhance chemosensitivity in multiple cancer cells, including breast cancer cells and hematological neoplastic cells [28, 29]. This establishes a chemosensitivity inhibition role of RBP1. Therefore, the negative correlation between PPARG and RBP1 supports the enhancement effect of PPARG on chemosensitivity.

However, we also noticed that PPARG presented a weak negative correlation with SELENBP1 (RHO = −0.24), which has been shown to increase the chemosensitivity of gastric cancer cells [30]. This may add to the explanation of the variation of PPARG in the CSP group of HSCC patients.

This study has several limitations that need further work. First, although we employed two independent datasets, both of them had small sample sizes. More studies with a larger sample size should be conducted to validate the findings of this study. Second, the identified coexpression factors of PPARG lack replication in other studies regarding their relation to chemosensitivity, which needs further validation.

## 5. Conclusion

Our results support the previous finding that PPARG expression was linked to chemosensitivity in HSCC patients. However, both increased and reduced PPARG expression could happen in chemotherapy-sensitive patients, which may be influenced by factors including the TNM stage.

## Figures and Tables

**Table 1:** Clinical data of HSCC patients for GSE85608.

Subject ID	Age	TNM stage	Histologic differentiation degree	Chemotherapy response	Gender
S1	69	T4aN2M0	Moderately differentiated	PR	Male
S2	62	T4aN2M0	Well differentiated	PR	Male
S3	69	T4N1M0	Poorly differentiated	PR	Male
S4	49	T3N2M0	Moderately differentiated	PR	Male
S5	60	T4bN2M0	Moderately differentiated	PR	Male
S6	69	T4aN0M0	Moderately differentiated	PR	Male
S7	44	T2N2M0	Poorly differentiated	PR	Male
S8	53	T4aN0M0	Well differentiated	PR	Male
S9	49	T4aN2M0	Moderately differentiated	PR	Male
S10	44	T4aN2M0	Poorly differentiated	PR	Male
S11	60	T3N1M0	Moderately differentiated	CR	Male
S12	48	T4bN2M0	Well differentiated	PR	Male
N1	65	T4aN2M0	Well differentiated	SD	Male
N2	45	T2N3M0	Moderately differentiated	PD	Male
N3	57	T4bN3M1	Well differentiated	SD	Male
N4	69	T3N2M0	Well differentiated	SD	Male
N5	71	T4aN2M0	Poorly differentiated	SD	Male
N6	43	T4bN2M1	Poorly differentiated	SD	Male
N7	69	T2N1M0	Well differentiated	SD	Male
N8	71	T4aN0M0	Well differentiated	SD	Male
N9	43	T4aN2M0	Moderately differentiated	SD	Male

Note: CR (complete response): disappearance; confirmed at 4 weeks; PR (partial response): 50% decrease; confirmed at 4 weeks; SD (stable disease): neither PR nor PD criteria are met; PD (progressive disease): 25% increase; no CR, PR, or SD documented before a progressed disease.

**Table 2:** Clinical data of HSCC patients for GSE85607.

Subject ID	Gender	Age	TNM stage	Histologic differentiation degree	Chemotherapy response
S1	Female	71	T2N0M0	Poorly differentiated	PR
S2	Male	68	T2N0M0	Well differentiated	PR
S3	Male	55	T4aN0M0	Well differentiated	PR
S4	Male	68	T3N0M1	Moderately differentiated	PR
S5	Male	58	T3N0M0	Well differentiated	PR
S6	Male	52	T2N0M0	Well differentiated	PR
S7	Male	56	T2N0M0	Well differentiated	PR
N1	Male	58	T4aN3M0	Poorly differentiated	SD
N2	Male	61	T3N2M0	Poorly differentiated	SD
N3	Male	56	T1N0M0	Well differentiated	SD
N4	Male	59	T3N2M0	Moderately differentiated	PD

Note: CR (complete response): disappearance; confirmed at 4 weeks; PR (partial response): 50% decrease; confirmed at 4 weeks; SD (stable disease): neither PR nor PD criteria are met; PD (progressive disease): 25% increase; no CR, PR, or SD documented before a progressed disease.

## Data Availability

The data in our study are available from the corresponding author upon reasonable request.
